# Atmospheric and Marine Corrosion of PEO and Composite Coatings Obtained on Al-Cu-Mg Aluminum Alloy

**DOI:** 10.3390/ma13122739

**Published:** 2020-06-17

**Authors:** Vladimir S. Egorkin, Ivan M. Medvedev, Sergey L. Sinebryukhov, Igor E. Vyaliy, Andrey S. Gnedenkov, Konstantine V. Nadaraia, Nikolaj V. Izotov, Dmitriy V. Mashtalyar, Sergey V. Gnedenkov

**Affiliations:** 1 Institute of Chemistry, Far Eastern Branch of the Russian Academy of Sciences, 690022 Vladivostok, Russia; egorkin@ich.dvo.ru (V.S.E.); sls@ich.dvo.ru (S.L.S.); igorvyal@gmail.com (I.E.V.); asg17@mail.com (A.S.G.); nadaraiakv@mail.ru (K.V.N.); nikolaj.izotov@mail.ru (N.V.I.); madiva@inbox.ru (D.V.M.); svg21@hotmail.com (S.V.G.); 2 All-Russian Scientific-Research Institute of Aviation Materials, 105005 Moscow, Russia

**Keywords:** plasma electrolytic oxidation, Al alloy, atmospheric corrosion, marine corrosion

## Abstract

Wrought Al-Cu-Mg aluminum alloy (D16) was treated by bipolar plasma electrolytic oxidation to create a base plasma electrolytic oxidation (PEO)-coating with corrosion protection and mechanical properties superior to bare alloy’s natural oxide layer. Additional protection was provided by the application of polymer, thus creating a composite coating. Electrochemical and scratch tests, scanning electron microscopy, energy-dispersive X-ray spectroscopy, X-ray diffraction studies were performed. Degradation of coatings in the marine atmosphere and seawater was evaluated. The composite polymer-containing coating provided better corrosion protection of aluminum alloy compared to the PEO-coating, although seawater affected both. During the atmospheric exposure, the PEO-coating provided reasonably good protection, and the composite coating showed excellent performance.

## 1. Introduction

The protection of structures in the marine environment is one of the major challenges in corrosion science. Aluminum alloys can be used in applications where weight efficiency is critical, such as high-speed boats, for offshore drilling pipes [1] or as a coating for protection of steel structures. Nowadays, Thermally Sprayed Aluminum (TSA) coatings are widely employed to protect steel components from corrosion in the marine environment [1,2,3,4]. However, 45% of the damage in coatings by mechanical stresses is mainly generated during transport and erection operations [5] due to poor tribological properties of bare aluminum alloys: low load-bearing capacity and low abrasion resistance [6]. Aluminum alloys with copper as the primary alloying element are of particular interest due to their high strength to weight ratio and good fatigue resistance. However, these alloys are often highly susceptible to localized corrosion in chloride-containing media, so protective coatings should be applied when they are used in corrosive environments.

Nowadays plasma electrolytic oxidation (PEO) is a very popular oxide growth technique, which is considered to be the most promising for light alloys. PEO is an environmental-friendly surface modification technique as alkaline electrolytes are used. The PEO process has many advantages compared to the conventional anodizing method [7,8,9,10]. Polymer coatings, especially the polytetrafluoroethylene, cannot be applied directly to the alloy because of lack of adhesion; surface pretreatment is required. PEO coating with its highly developed surface provides an excellent base for polymer application [11]. The PEO surface modification followed by the formation of polymer-containing composite coatings, as previously studied by Gnedenkov [12,13,14] for magnesium alloys and R. Zhang [15] and C. Lu [16] for aluminum alloy 2024, can provide wear properties and corrosion protection superior to natural oxides, formed on aluminum alloys [17]. The vast majority of electrolytes widely used for PEO of Al and Al alloys are based on alkaline solutions (NaOH or KOH) with additions of phosphates [18] and/or silicates [6,19,20,21,22,23,24,25,26,27,28,29,30], and aluminates [26,31].

Although novel aluminum alloys with improved mechanical properties are developed, AA 2024 (Al-Cu-Mg alloying system) remains major construction material. The galvanic corrosion of AA 2xxx aluminum alloys in chloride-containing environments remains an important challenge. In particular, AA 2024 alloys are highly susceptible to localized corrosion, due to galvanic coupling formed between the Al matrix and the Mg and/or Cu based intermetallic particles [32]. Most of the reported PEO corrosion protection studies on AA 2024 alloy were performed using electrochemical impedance spectroscopy (EIS) and potentiodynamic polarization [18,23]. Wen et al. [28] immersed the AA 2024 alloy substrates with PEO-coating in 3% NaCl solution up to 168 h and studied surface morphology and performed EDS analysis of corrosion products. It was shown that after 24 h of immersion the corrosive species penetrated the defects in the coating and the localized corrosion under the coating took place. Shchedrina et al. [19] subjected AA 2024 alloy with 80 µm PEO-coating to 336 h salt spray test and no corrosion spots were observed, although no microstructural or electrochemical investigations were performed to support this finding.

Accelerated tests, such as neutral salt spray, are widely used to evaluate the protective properties of coatings. During the atmospheric exposure, specimens are subjected to the synergetic influence of many environmental factors: hydrothermal cycles, precipitations, deposition of corrosive species [33]. Marine immersion also provides a variety of environmental variables that are hard to replicate in lab conditions, especially the influence of microorganisms on the tested coating [3,34]. Therefore, accelerated tests are helpful for quick assessment of corrosion protection, but validation by exposure tests in a real marine environment is required [3].

The current work aims to investigate protective properties of PEO and composite coatings (CC) on D16 aluminum alloy (Al-Cu-Mg system, AA 2024 equivalent [35]) during the short time exposure to the marine atmosphere and seawater (marine immersion).

## 2. Materials and Methods

### 2.1. Samples Preparation

Unclad 2 mm Al-Cu-Mg (D16) aluminum alloy (1.6 wt. % Mg, 4.9 wt. % Cu, 0.8 wt. % Mn, 0.2 wt. % Si, 0.4 wt. % Fe, 0.2 wt. % Zn, Al balance) sheeting was cut to 50 mm × 50 mm rectangular samples. The samples were wet ground with SiC abrasive papers subsequently with different grits from 240# to 1200# and rinsed in distilled water before oxidation to achieve a standardized surface, which should provide more reliable electrochemical and adhesion test results, even though the PEO process inevitably produces a rough surface.

The following solution, prepared using deionized water, was used as an electrolyte for the PEO: 0.6 g/L NaF, 5 g/L C_4_H_4_O_6_K_2_∙0.5H_2_O, 10 g/L Na_2_MoO_4_∙2H_2_O, 10 g/L Na_2_B_4_O_7_∙10H_2_O, 10 g/L Na_3_PO_4_∙12H_2_O. The use of C_4_H_4_O_6_K_2_∙0.5H_2_O provided an increase in the duration of microplasma discharges and inclusion of carbon (in the form of different carbides) in the growing PEO-layer. Due to the high oxidizing ability of fluoride ions, NaF leads to the activation of the oxidation of aluminum at the beginning of PEO. By increasing the electrical conductivity of the electrolyte, it contributes to an increase in its dissipative ability, which, as a rule, provides a stable voltage supply to the sample and allows the formation of thicker and denser PEO-layers. Na_2_B_4_O_7_∙10H_2_O served to buffer the electrolyte in order to avoid lowering the pH of the solution, which can lead to the destruction of complex anions, and also contributed to reducing the porosity of coatings. Na_3_PO_4_∙12H_2_O provided the alkaline pH of the electrolyte to the alkaline zone during its preparation for oxidation. Phosphate anions are responsible for the formation of a barrier inner layer and facilitate its protective properties. Na_2_MoO_4_∙2H_2_O contributed to the improvement of the decorative characteristics of coatings, as well as the synthesis of molybdenum carbides, which provided a significant increase in the microhardness of PEO-coatings.

This electrolyte grants the possibility of formation of the smooth layers on pure aluminum and a wide range of its alloys including Cu-, Mg-, Zr-doped with stable characteristics without the necessity to change the electrolyte’s composition/concentration and electrical conditions. To prevent electrolyte overheating H150-3000 Chiller Smart device (LabTech Inc., Hopkinton, MA, USA) was used to maintain electrolyte temperature at 20 °C during the PEO process.

The PEO-coating formation process was performed using the bipolar pulse mode. The frequency was equal to 300 Hz. The duration ratio of the anodic and cathodic pulses was equal to 1. The voltage was escalated from 30 to 360 V at 65 V min^−1^ rate during the anodic period. Then the voltage climb rate was decreased to 2.5 V min^−1^ for 30 min. At the end of the PEO process, the voltage was 420 V.

Constant 100 mA cm^−2^ current density was maintained during the cathodic period. The electrical power for the PEO process was supplied by the thyristor rectifier linked to a PC-controlled voltage and current regulator. After the oxidation, the coated samples were rinsed with distilled water and dried in air at the ambient room temperature.

To form a composite coating (CC) the polymer was applied atop the PEO-coating to provide sealing of the pores. Superdispersed polytetrafluoroethylene (SPTFE) powder isopropanol suspension (15 wt. %) was used for the formation of composite layers by the dip-coating method. After 10–15 s of dipping, samples were placed for 5 min in the oven, heated up to 315 °C, which resulted in the melting of the polymer, providing efficient sealing of pores. After the heat treatment samples were cooled at room temperature and the application of polymer, heat treatment was repeated two more times to obtain a uniform well-sealed composite layer.

### 2.2. Electrochemical Measurements

VersaSTAT MC 4-channel potentiostat/galvanostat (Princeton Applied Research, USA) was used for electrochemical measurements. Neutral (pH = 7) 3% NaCl solution at room temperature was used as an electrolyte. Three-electrode setup with the platinum-coated niobium mesh counter electrode and Ag/AgCl_sat.KCl_ reference electrode (E_vs.NHE_ = + 0.201 V) was used. The working electrode area (exposed sample surface) was 1 cm^2^. To achieve a steady state, required for measurements, the samples were kept for 150 min in the cell, filled with electrolytes.

The potentiodynamic polarization was performed at 1 mV/s scan rate; specimens were polarized in the anodic direction from −250 mV to +1.5 V vs. *E*_c_. VersaStudio software (Princeton Applied Research, Oak Ridge, TN, USA) was used to control the measurements and provide data acquisition. Potentiodynamic data were fitted using the Levenberg-Marquardt (LEV) method and to obtain corrosion potential *E*_c_, corrosion current density *j*_c_, cathodic and anodic Tafel slopes of polarization curve. Separate linear polarization resistance (LPR) measurements in the *E*_c_ ± 20 mV range were used to measure the polarization resistance *R*_p_ *=* Δ*E/*Δ*j*. Data were fitted using CView software v. 3.5f (Scribner Associates Inc., Southern Pines, NC, USA).

**Figure 1:**
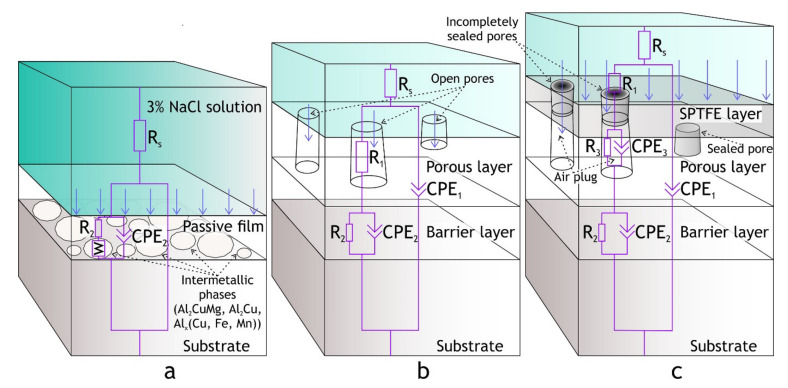
Equivalent electrical circuits used for the impedance spectra analysis: (**a**) Al-Cu-Mg aluminum alloy, (**b**) plasma electrolytic oxidation (PEO)-coating, (**c**) composite coating.

Electrochemical impedance spectroscopy measurements were conducted at open circuit potential in the frequency range from 0.1 to 0.02 Hz (logarithmic sweep) by application of 10 mV rms sine-wave electrical signal. The impedance spectra were fitted using the equivalent electrical circuits (EEC) shown in Figure 1. Constant phase elements (CPE) were used in the EECs instead of the capacitors to describe the imperfect capacitance in heterogeneous systems such as PEO and composite coatings. The CPE impedance is calculated by Equation (1):

(1) $$Z_{CPE}=\frac{1}{{Q(j\omega )}^{n}}$$

where *ω* is the angular frequency (*ω = 2πf*), *j* is an imaginary unit, *n* and *Q* are the exponential coefficient and the frequency-independent constant, respectively.

For fitting the impedance spectra of PEO-coating the conventional two R-CPE circuits were used, in which the *R*_1_-CPE_1_ circuit describes the properties of the porous outer layer and R_2_-CPE_2_ circuit corresponds to the poreless inner layer. Due to the sealing of the pores with a polymer in composite coatings third R-CPE circuit is introduced to the EEC to describe composite layers including polymer plugs in the pores (Figure 1). Fitting of the impedance spectra by the EECs was performed using ZView v. 3.5f (Scribner Associates, Inc., Southern Pines, NC, USA).

We have performed electrochemical tests on at least 3 specimens for each reported value. The calculated values of corrosion parameters have the root mean square deviation of less than 5%. The standard deviation was not included in tables to improve the readability of tables, especially long ones.

### 2.3. Surface Morphology and Phase Analysis

The surface and cross-sectional morphology of the coatings were observed using scanning electron microscope Zeiss EVO 40 (Carl Zeiss Group, Jena, Germany) in the Far Eastern Center for Electron Microscopy of National Scientific Centre of Marine Biology FEB RAS (Vladivostok, Russia). Micrographs of the surface of PEO-coatings were obtained using a secondary electron detector, and cross-sections using a back-scattered electron detector at an accelerating voltage of 20 kV.

To avoid the excessive charging of non-conductive coatings, causing image distortion, thin Cr and Au films were deposited on the samples’ surface. Cr and Au peaks were excluded from EDX spectra. X-MaxN 80 Silicon Drift Detector (Oxford Instruments plc, Abingdon, UK) was used for EDX. As can be seen from the figures, the EDX analysis was performed at regions that do not have the same area. The choice of analysis area has to be a compromise between the large area for averaging of data and a small area for localization of a particular part of a coating. Unfortunately, it was not possible to choose the same area size for all measurements due to the inhomogeneity and complex morphology of the studied coatings. The EDX spectra were normalized automatically during the analysis by Aztec software. Bruker D8 ADVANCE X-ray diffractometer (XRD) was used to study the phase composition of coatings. CuKα radiation source was operated at 30 kV accelerating voltage, 30 mA current, 2 s exposure time, 0.028 2θ step size, 5–80° 2θ range.

### 2.4. Scratch Adhesion Test

CSM REVETEST (CSM Instruments, Peuseux, Switzerland) macro scratch tester, equipped with the Rockwell diamond indenter, was used for scratch adhesion tests. The sample was moved at 4.99 mm/min speed while the indenter was fixed still. Scratch test was started at 0.9 N load and finished at 20 N, resulting in a 10.59 N/min loading rate. The normal and tangential forces, indenter penetration depth and level of acoustic emission were measured during the test. Critical loads *L*_c2_, *L*_c3_ were identified using scratch image analysis, acoustic emission peaks and changes in the slope of forces. Before the test, the surface profile was measured on each sample to reduce the error in the determination of the indenter penetration depth under loading.

### 2.5. Corrosion Tests

Uncoated aluminum alloy, samples with PEO and composite coatings were exposed at the atmospheric corrosion stand (Corrosion exposure station) of Institute of Chemistry Far Eastern Branch of Russian Academy of Sciences (ICH FEB RAS) The place is classified as a marine environment with medium (C3 ISO 9223:2012) atmospheric corrosivity [36]. The samples were exposed at an angle of 45° to the horizon on racks about 20 m away from the seashore.

The same set of samples was immersed in the natural seawater (Sea of Japan). The average seawater salinity is 33–35%. The test rack was fixed at the depth of 1 m from the surface and 1 m from the sea bottom.

The samples were withdrawn from the test site after 7, 14 and 30 days of exposure. The samples were rinsed by water, dried, weighed using analytical balance Shimadzu AUW120D (Shimadzu Corporation, Kyoto, Japan). Corrosion products from uncoated samples were removed by pickling in HNO_3_ for 5 min at room temperature; afterward, the samples were reweighted to calculate mass losses.

## 3. Results and Discussion

### 3.1. Coatings Properties

#### 3.1.1. Surface Morphology and Phase Analysis

**Figure 2:**
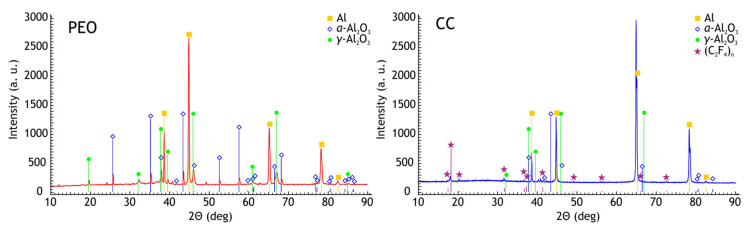
XRD spectra of PEO and composite coatings.

The X-ray diffraction patterns of PEO-coating and composite coating are shown in Figure 2. Due to the small reflectivity of the coating, strong aluminum diffraction peaks are detected. The α-Al_2_O_3_ and γ-Al_2_O_3_ are typical phases in PEO-coatings on aluminum alloys. The concentration of the α-Al_2_O_3_ phase for such coating is typically low and often is not detected by the XRD. Additionally, the polytetrafluoroethylene (C_2_F_4_)_n_ peaks are present in the sample with composite coating XRD spectra.

**Figure 3:**
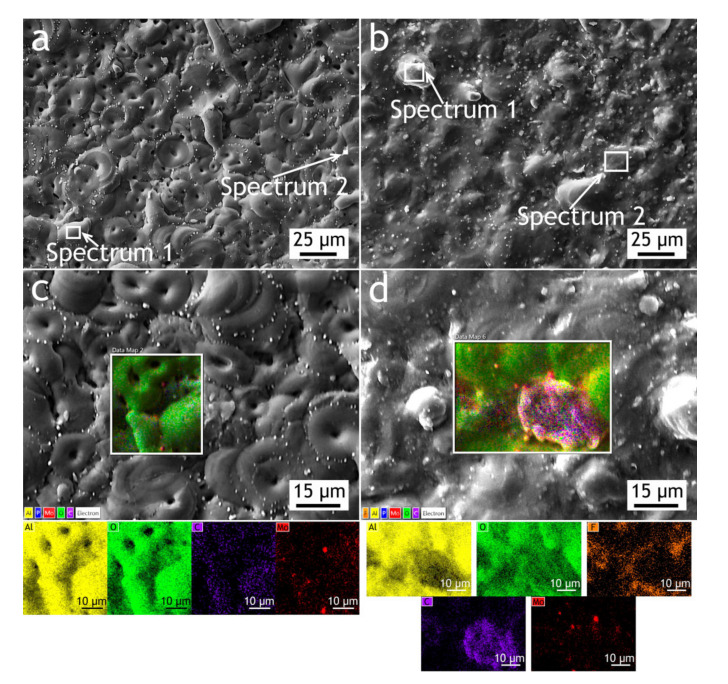
SEM images and EDX maps of the PEO-coating—(**a**,**c**) and composite coatings (CC)—(**b**,**d**).

**Figure 4:**
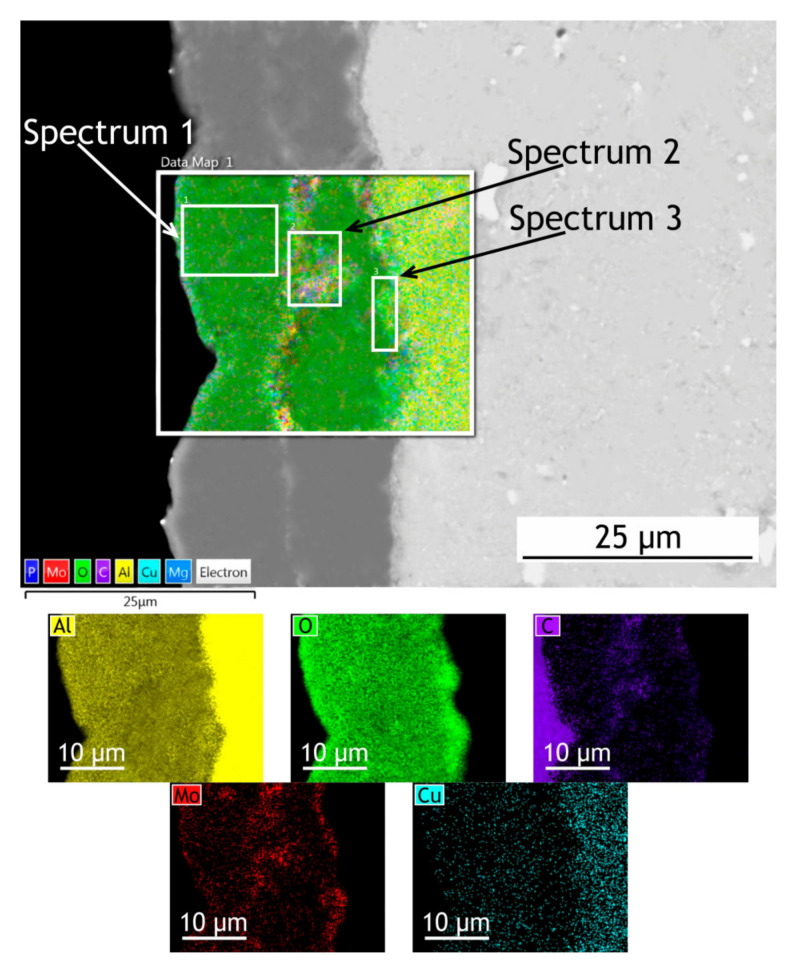
SEM image and EDX maps of the PEO-coatings’ cross-section.

**Figure 5:**
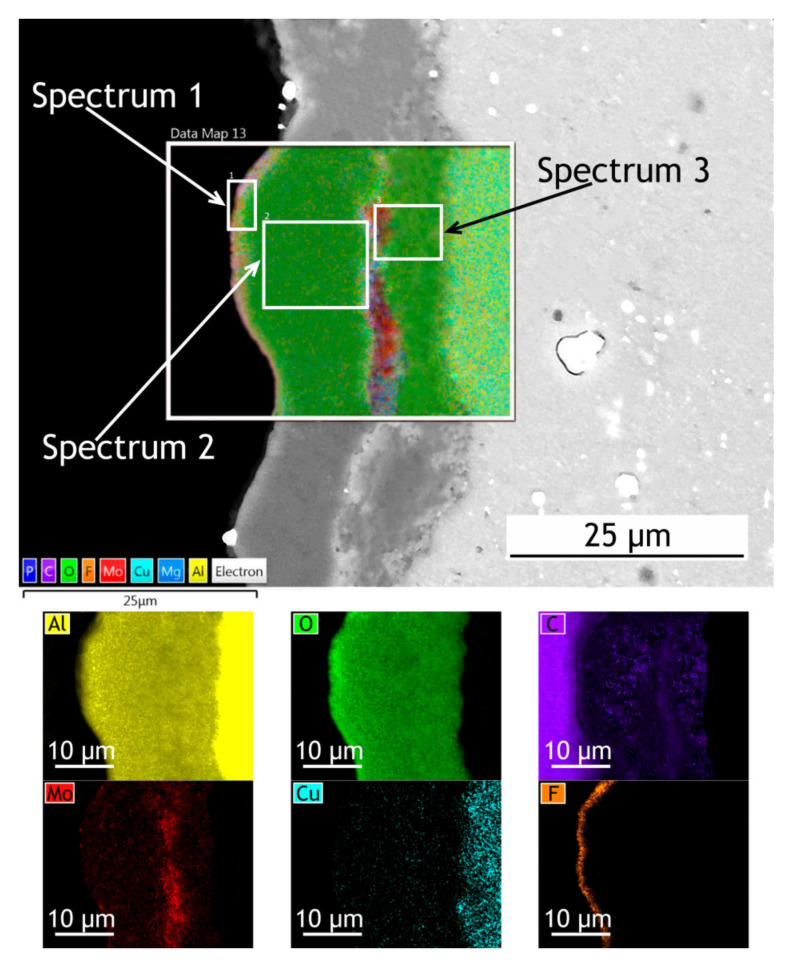
SEM image and EDX maps of the CC cross-section.

The surface, cross-sectional morphology, as well as elemental distribution in PEO and composite coatings, are shown in Figure 3, Figure 4 and Figure 5. The chemical composition of the coatings obtained by PEO in complex electrolytes is not homogeneous. For example, in [37] the coating on Mg alloy consisted mostly of Mg_2_SiO_4_ but MgF_2_ in the dense inner layer was responsible for an increased level of barrier properties. In [38] Quintero et al. investigated coatings were obtained in three different phosphate-containing electrolytes. All the coatings exhibited inhomogeneous chemical compositions, which resulted in differences in corrosion behavior. In our case, the chemical composition of the coating enabled a significant decrease in the corrosion currents. It is well known that plasma discharge on the surface of the alloy subjected to the PEO process causes the formation of discharge channels in the oxide layer. It can be seen in Figure 3a,c that the formed oxide layer is porous with a lot of pores 1–3 μm in diameter. The pores are significantly reducing the corrosion protection [39] as the corrosive media penetrates pores and may initiate corrosion processes at the coating/substrate interface. On the other hand, a highly porous oxide layer serves as a good substrate for polymer application, providing high adhesion between the polymer and oxide layers. After the formation of the polymer composite coating, pores are almost completely sealed with polytetrafluoroethylene (Figure 3b,d).

If we pay attention to the surface morphology of the coating, we can conclude that it is composed of two main units: pancake structures with discharge channels in the center (which are common for the PEO-layers [40,41,42]) and small particles with 0.75–1 µm dimensions. To reveal the possible discrepancies in the chemical composition of these units, the EDX spectra were collected at the regions corresponding to the pancake (spectrum 1) and the particle (spectrum 2) Figure 3, Table 1. It should be noted that the particles are distributed almost equidistantly from the pore mouths forming overlapping circles on the surface of the coatings. It is obvious therefore that their appearance is caused not by the precipitation or deposition processes, but by plasma electrolytic oxidation. According to the EDX data, they consist predominantly of molybdenum assumingly as molybdenum oxide. It might be suggested that it is such conditions in the molted coating’s material near discharge channels during the PEO process that lead to predominant concentration and diffusion of Mo to the surface during the cooling and solidification. According to the EDX analysis (Table 1), all the elements from the electrolyte solution are incorporated into the oxide layer during the PEO process. The presence of carbon in the PEO-coating’s composition is explained by C_4_H_4_O_6_K_2_∙0.5H_2_O as the constituent of the electrolyte. As it was shown in [43], addition of potassium tartrate (C_4_H_4_O_6_K_2_∙0.5H_2_O) into electrolytes containing aqueous solutions of sodium hexafluorinealuminate (Na_3_AlF_6_), sodium fluoride (NaF), and sodium hydroxide (NaOH) during PEO-treatment of aluminum alloys results in the appearance of carbon in the coating’s chemical composition. The presence of the fluorine and the higher carbon content in the composite coating in comparison with the PEO-layer is a result of sealing with polytetrafluoroethylene.

Since X-ray diffraction analysis has rather low sensitivity and, according to energy dispersive X-ray analysis data, the molybdenum content of the coatings did not exceed 5% (Table 1 and Table 2), no reflections from molybdenum-containing phases were detected in the X-ray diffraction patterns of the coatings under investigation. Nevertheless, it was found [44] that carbides in the coating are presented in amorphous form. The presence of Al_2_Mo_3_C phase could be revealed by XRD and XPS after annealing at 600 °C [44], moreover according to XPS the possible presence of Mo_2_C and MoC could not be ruled out. Formation of the carbides is not a unique situation, in [45] the carbides were found in the PEO-coating on titanium alloy in accordance with the XPS data that the coatings formed in this electrolyte contain molybdenum aluminum carbide (Al_2_Mo_3_C). The formation of such a phase could be explained by the fact that under conditions of plasma electrolytic oxidation, the temperature in discharge channels on the surface being processed may considerably exceed 3000 K [46]. Such conditions intensify water thermolysis, leading to the formation of a predominantly reducing atmosphere [47]. In addition to thermolysis, an important role in water decomposition will be played by water electrolysis during PEO. The cathode phase of bipolar (anode-cathode) mode involves hydrogen formation at the electrode according to the electrochemical reaction 2H_2_O + 2e^−^→H_2_ + 2OH^−^. Thus, a reducing hydrogen atmosphere may lead to the formation of molybdenum carbides. Given that the coatings contain a considerable amount of carbon (Table 1 and Table 2), the formation of Mo_2_C and MoC is also possible.

To investigate how elements are distributed across the coating thickness EDX maps (Figure 4 and Figure 5) are provided and Table 2 summarizes the elemental composition of the coating’s top, middle, bottom parts. Copper is being depleted from the alloy near the coating-alloy interface: Cu content is below 1.2% in these areas while in a bulk alloy it can reach 4.9%. In Figure 5 the fluorine content in the surface layer of CC indicates the presence of a continuous polytetrafluoroethylene layer. The thickness of the base PEO-layer can vary, as shown in figures with the cross-sections, which is common for PEO coatings and assumingly can lead to local breakdowns where the coating is thin or more porous during corrosion tests.

#### 3.1.2. Electrochemical Measurements

**Figure 6:**
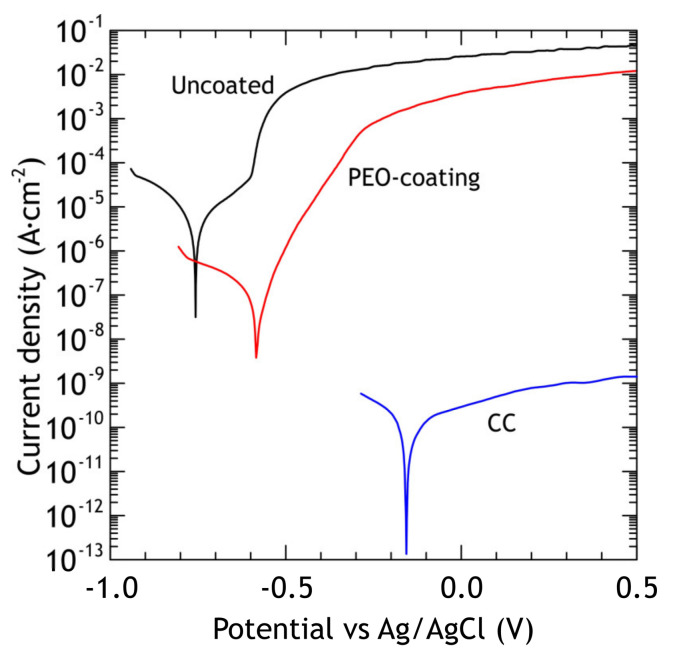
Potentiodynamic polarization curves obtained in 3% NaCl for the bare alloy, PEO and composite coatings.

**Figure 7:**
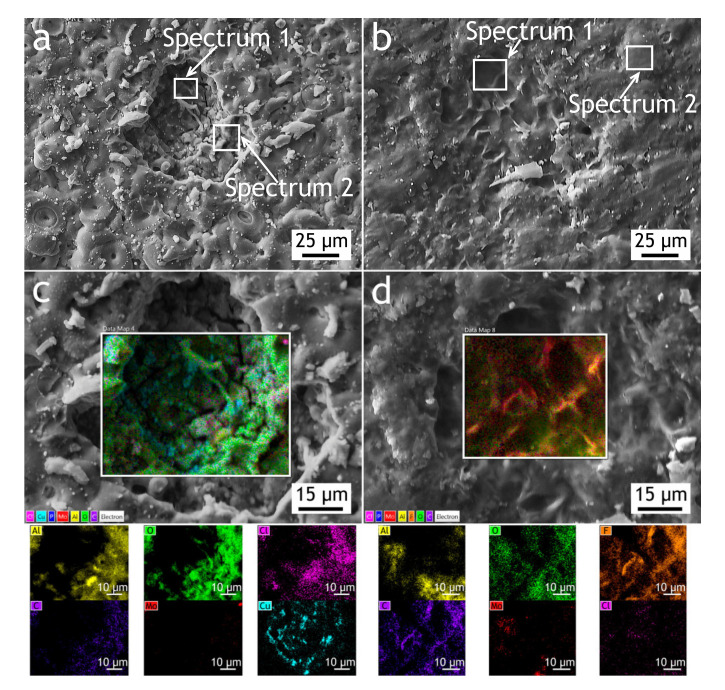
SEM images and EDX maps for the PEO-coating—(**a**,**c**) and CC—(**b**,**d**) after potentiodynamic polarization test performed in 3% NaCl.

According to the reported researches, PEO-coatings obtained in different electrolytes on aluminum alloys are significantly more chemically/electrochemically stable compared to the substrate material and the protection efficiency is strongly dependent on the porosity of the PEO-coatings [28,48]. The corrosion process for such coatings is associated with morphological defects, which are formed during the PEO-process. Aluminum oxide, aluminum phosphate, and aluminum-molybdenum carbide in the coating are more stable compounds in sodium chloride solution in comparison with aluminum alloy. During the electrochemical polarization, the corrosion process occurs through the defects of the poreless sublayer adjacent to the substrate. Corrosion products could lead to the appearance of mechanical stresses in the coating resulting in the partial destruction of the PEO-layer. The potentiodynamic polarization curves of PEO and composite coatings and bare alloy are shown in Figure 6. Using CView software, the anodic/cathodic Tafel constants, corrosion current density, corrosion potential and polarization resistance (from separate experimental data) were calculated and presented in Table 3. The corrosion potential of PEO-coating is −583 mV and it is 174 mV nobler compared to the bare alloy’s corrosion potential. The corrosion current density for the PEO-coating is one order of magnitude lower compared to bare alloy’s corrosion current. Composite coating corrosion potential is equal to −160 mV and this value is 597 mV higher than the bare alloy’s corrosion potential and the corrosion current density is 4 orders of magnitude lower compared to the bare alloy’s corrosion current density. Therefore, sealing PEO-coating’s pores with polytetrafluoroethylene results in better corrosion protection properties. Corrosion current density and polarization resistance for CC are 1.1 × 10^−10^ A·cm^−2^ and 2.2 × 10^8^ Ω·cm^2^, respectively. Potentiodynamic polarization (anodic stage) causes damage to PEO-coating and underlying aluminum alloy (Figure 7a,c), resulting in the appearance of pits, which reach the substrate. The pitting zone is enriched with copper (Table 4). Although there are some cavities at the CC coating surface, they do not penetrate through the whole coating thickness (Figure 7b,d) due to their high isolating properties: current density is below 10^−9^ A·cm^−2^ even at 1.5 V.

In aluminum alloys, coarse intermetallic particles, containing considerable amounts of Cu, Fe, Mn, and Si behave mainly cathodically, while phases with significant levels of Mg act as anodes undergoing dealloying (Al and Mg removal) leaving copper-rich remnants that may behave cathodically [32]. Due to large potential difference between S-phase (Al_2_CuMg) and other intermetallic particles (Al_7_Cu_2_Fe, Al_20_Mn_3_Cu_2_, Mg_2_Si, etc.), the current densities are high [32,49] and local dissolution of Al and Mg in S-phase occurs, leading to local copper enrichment and further localized corrosion is a well-known phenomenon for Al-Cu-Mg alloys [50,51,52,53,54]. For the multi-phase intermetallic particles consisting of both θ-phase (Al_2_Cu) and S-phase, de-alloying might occur preferentially at the S-phase and the de-alloying of the θ-phase particles initiated in the regions surrounding the S-phase [55]. In Figure 7a,c it can be seen that the corrosion test site is enriched in copper, seemingly due to copper redeposition, which leads to further corrosion propagation [56,57]. In accordance with [58,59] the main corrosion product for Al-Mg-Cu alloys is Al_2_O_3_/Al(OH)_3_. However, the results of S-phase investigation [60] using model Al-Mg-Cu sample show that strong local anodic activity was detected on Mg part of the specimen. This indicates the probable Mg(OH)_2_ formation. Nevertheless, due to the low concentration of Mg as compared to Al in the sample composition and unfavorable pH of the solution (due to Al dissolution), the content of Mg(OH)_2_ component was not detected using the surface analysis in the aforementioned studies.

The results of the work [61] show the possibility of CuO formation on the cathodic zones of the material and the probable Al(OH)_2_Cl formation on the sites of the sample with pH equals 4.7. In our study, the results of the EDX analysis (Figure 7) showed the Al and Cu presence in the pitting zone formed after potentiodynamic polarization.

**Figure 8:**
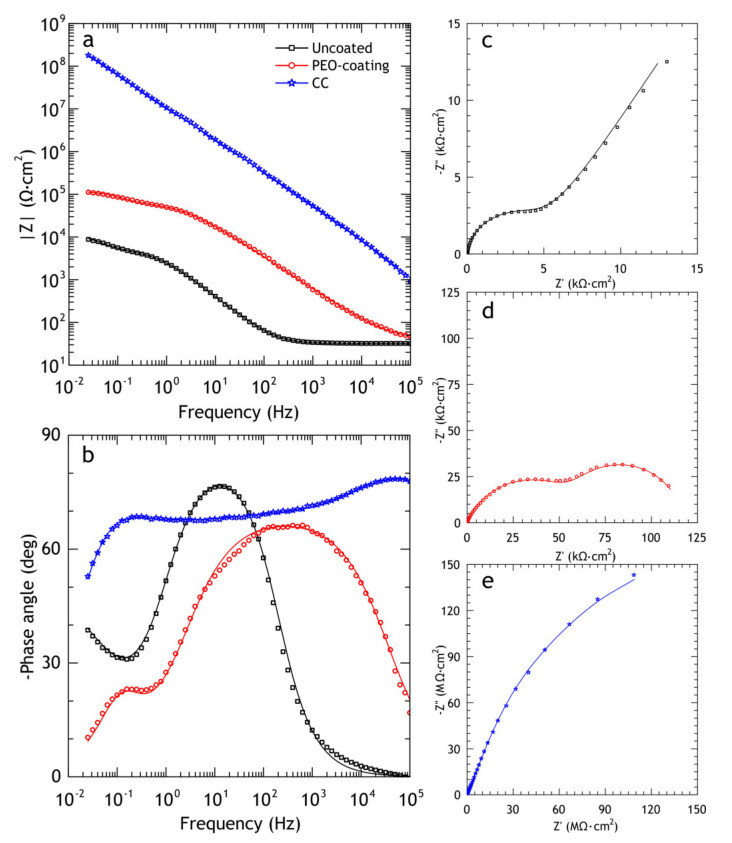
Impedance data obtained in 3% NaCl for the studied samples. Bode—(**a**,**b**) and Nyquist plots for the bare alloy (**c**), PEO (**d**) and composite (**e**) coatings.

The EIS spectra of bare alloys, PEO and composite coatings, are presented in the Bode and Nyquist plots in Figure 8. The application of PEO-coating resulted in a rise in impedance modulus (|Z|_f = 0.02 Hz_) by one order of magnitude compared to the bare alloy, while application of a polymer to PEO oxide layer resulted in more than four orders of magnitude higher impedance modulus compared to the uncoated alloy. Moreover, impedance modulus of the polymer-containing coating is higher than PEO-coating over an entire range of frequencies, indicating that the application of composite coating sufficiently increases the barrier properties of the coating. Phase angle plot (Figure 8b) shows that two time constants are present in PEO-coating impedance spectra; this corresponds to the two-layer structure common for PEO-coatings, which include dense poreless inner sublayer and porous outer layer. As for bare aluminum alloy, the impedance modulus and the presence of low-frequency time constant are typical for bare Al-Cu-Mg alloys exposed to NaCl aqueous solution. Zheludkevich [36] suggested that low-frequency time constant could be indicative of a diffusion-limited corrosion process. This part of the spectrum can be modeled using the porous bounded Warburg element, *W,* an impedance of which is, $Z=R_{W}\frac{\coth{\left(iT\omega \right)}^{P}}{{\left(iT\omega \right)}^{P}}$ where *i* is the imaginary unit, *ω* is the angular frequency of the AC signal, *T* = *L*^2^/*D* (*L* is the effective diffusion thickness, and *D* is the effective diffusion coefficient of the particle), 0 < *P* < 1 [62]. This element was also used by other researchers to model low-frequency impedance of Al-Cu-Mg alloy, immersed in NaCl solution [58,63]. Due to poor corrosion resistance, D16 alloy does not reach full passivation and the surface is prone to localized corrosion. Therefore, to model the impedance spectrum for the bare alloy we used the electrical equivalent circuit with one *R*_2_-CPE_2_ circuit, representing passive oxide film, coupled with *W* element (Figure 1a).

Composite coating impedance spectra is more complicated for analysis, and time constants are hard to separate due to strong overlapping. Based on the previous research by Gnedenkov [64], EEC with three *R*-CPE circuits (Figure 1c) was applied, which provided a reasonably good fit (average χ^2^ ≈ 0.04), which could not be achieved by the application of EEC with two *R*-CPE circuits. Impedance spectra were fitted using EECs in Figure 1 and corresponding values are presented in Table 5. Fitting curves (solid lines in Figure 8) were plotted using these parameters. The decrease in *Q*_1_ for CC coatings compared to PEO can be attributed to an increased thickness of the coating, and an increase in *R*_1_ and *R*_2_ is a result of partial sealing of the pores in the porous layer and defects in the inner oxide layer. High values of *R*_3_ represent the low conductivity of polymers inside the pores. The capacitive behavior over a wide frequency range indicates the high quality of sealing.

#### 3.1.3. Scratch Test

**Figure 9:**
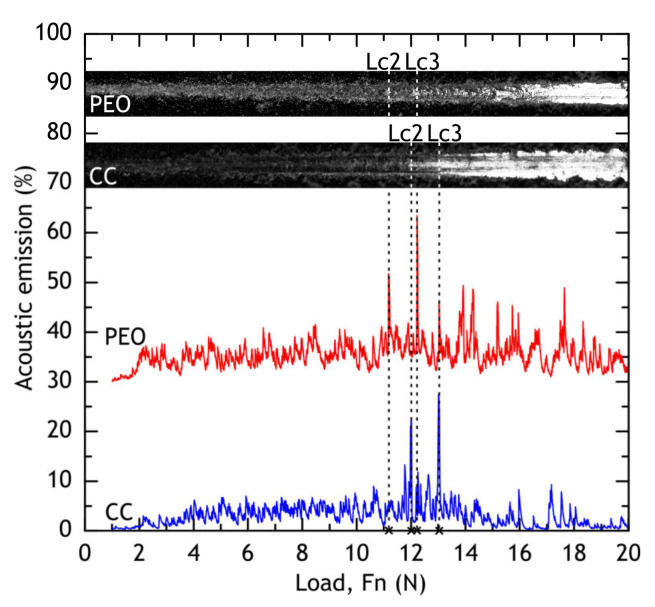
Micrograph of a full scratch, critical loads, and acoustic emission of PEO and composite coatings.

**Figure 10:**
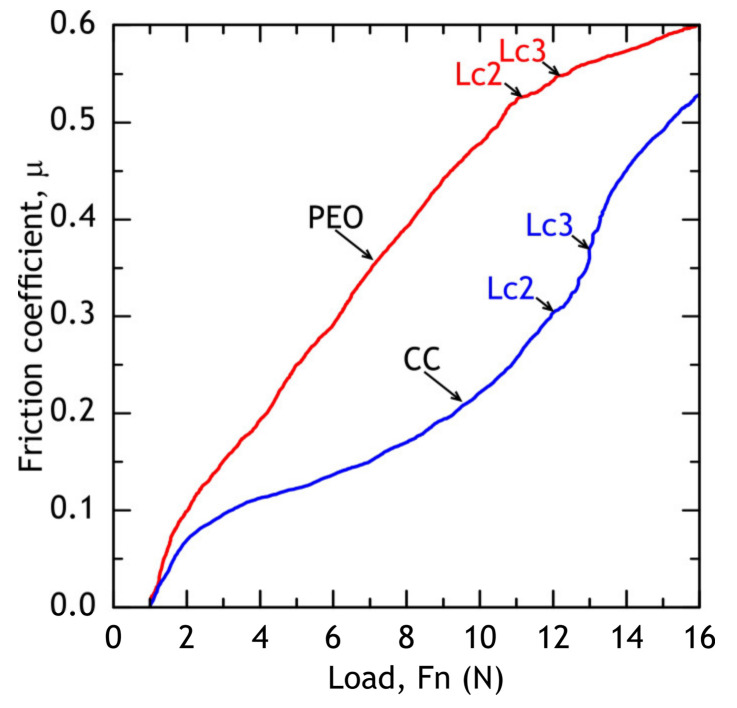
Evolution of friction coefficient during the scratch test of PEO and composite.

Scratch test was used to evaluate coatings’ adhesion to the substrate (alloy) and measure the friction coefficient of PEO and composite coatings. The micrograph of a full scratch and acoustic emission are shown in Figure 9. The dependence of the friction coefficient on the applied force during the scratch test is shown in Figure 10. In case of PEO-coating, the coating failure can be easily correlated with a significant change of a friction coefficient to applied force (*µ*/*F*_n_) slope, while for the composite coating failure is not as evident: even when the substrate is reached, the polymer works as a lubricant, providing a steady increase in friction force (see Figure 10). Using acoustic emission, image analysis and *µ* = *f*(*F*_n_) dependence, the critical normal loads (*L*_c_) were measured for PEO and composite coatings. For PEO-coating *L*_c2_ = 11.2 ± 0.1 N, *L*_c3_ = 12.1 ± 0.1 N, while composite coating provided slightly better scratch resistance: *L*_c2_ = 12.1 ± 0.1 N, *L*_c3_ = 12.5 ± 0.2 N. The composite coating provides a friction coefficient below 0.3 before the critical normal load *L*_c2_ is reached.

### 3.2. Atmospheric Corrosion

**Figure 11:**
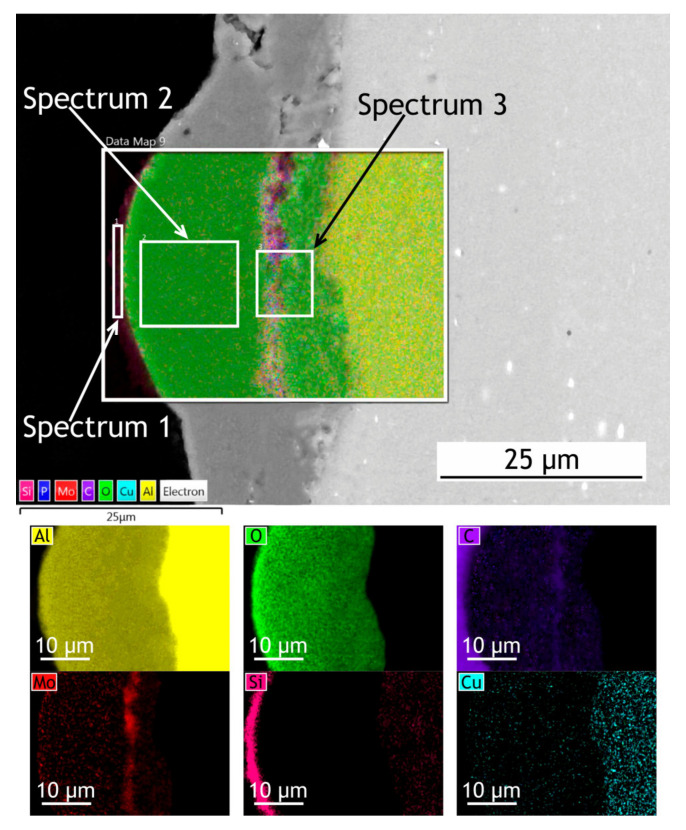
SEM image and EDX maps of the PEO-coating’s cross-section after 30 days of atmospheric exposure.

**Figure 12:**
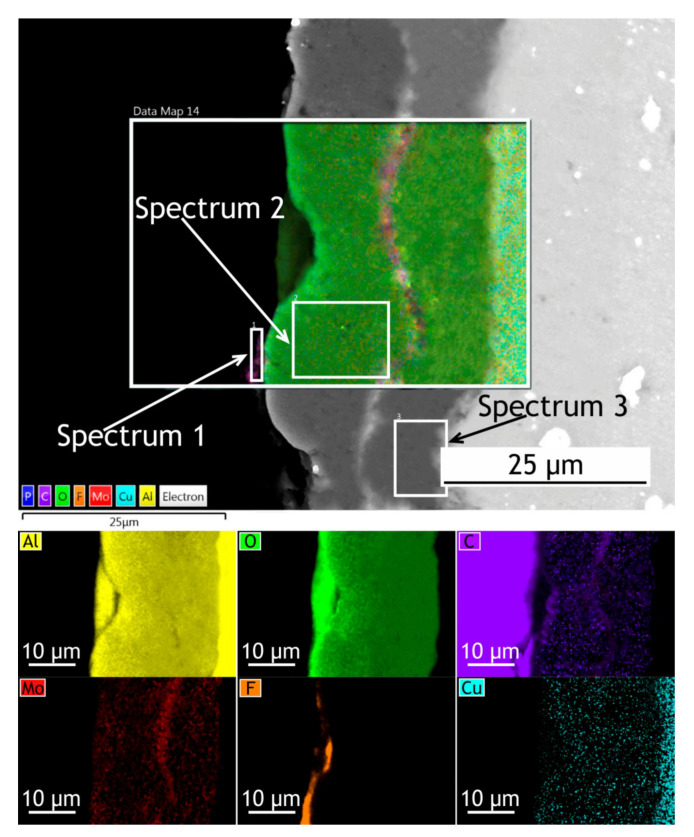
SEM image and EDX maps of the CC’s cross-section after 30 days of atmospheric exposure.

EDX maps of PEO and composite coatings’ cross-section after 30 days of atmospheric exposure are shown in Figure 11 and Figure 12. Table 6 summarizes the elemental composition of the coating’s top, middle, and bottom parts. Due to dust deposition during atmospheric exposure, silicon is present on the coating surface.

After atmospheric exposure of PEO-coating, we see little changes in the overall chemical composition (Table 6, Figure 11), compared to the specimen before exposure (Table 2). As for the composite coatings’ cross-section, we also do not see many changes (Figure 12); there is a distinct fluorine-containing layer, which means that polytetrafluoroethylene is present in the top layer of the coating. At the top of the coating, fluorine content is up to 20.9 wt. %. We see high molybdenum content (3.9–4.9 wt. %) for both PEO and composite coatings in the middle part of the coating. These results indicate that there is no degradation of the coating material during atmospheric exposure.

**Figure 13:**
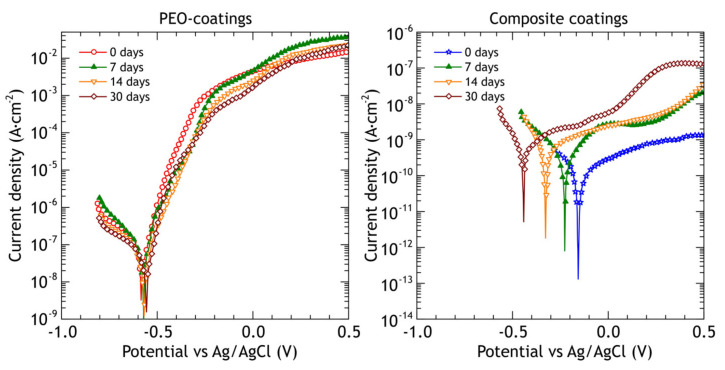
Potentiodynamic polarization curves obtained in 3% NaCl for PEO and composite coatings before and after 7, 14 and 30 days of atmospheric exposure.

The samples were withdrawn after 7, 14, 30 days of atmospheric exposure and electrochemical impedance spectra and potentiodynamic polarization curves were recorded on at least three different samples. Potentiodynamic polarization curves of PEO and composite coatings before corrosion testing and after 7, 14 and 30 days of atmospheric exposure are shown in Figure 13, and the calculated electrochemical parameters are presented in Table 7.

The slope *β*_a_ of the anodic branch of the polarization curve obtained for PEO-coating slightly decreases, indicating higher susceptibility to localized corrosion and coating failure under lower potentials, compared to reference coating. The values of corrosion potential *E*_c_ are slightly nobler, which may be attributed to corrosion products deposition on active corrosion sites. Corrosion current density *j*_c_ decreased after 30 days of exposure from 1.2 × 10^−7^ A·cm^−2^ to 3.9 × 10^−8^ A·cm^−2^, which can be explained by the same reasons as the corrosion potential change. The polarization resistance *R*_p_ behaves inversely, increasing from 1.8 × 10^5^ Ω·cm^2^ to 4.8 × 10^5^ Ω·cm^2^.

The composite coatings barrier properties remain very high during exposure tests. The observed increase in corrosion current density *j*_c_ with the corresponding decrease in polarization resistance values may be caused by a few point defects in the polymer layer due to UV-radiation.

**Figure 14:**
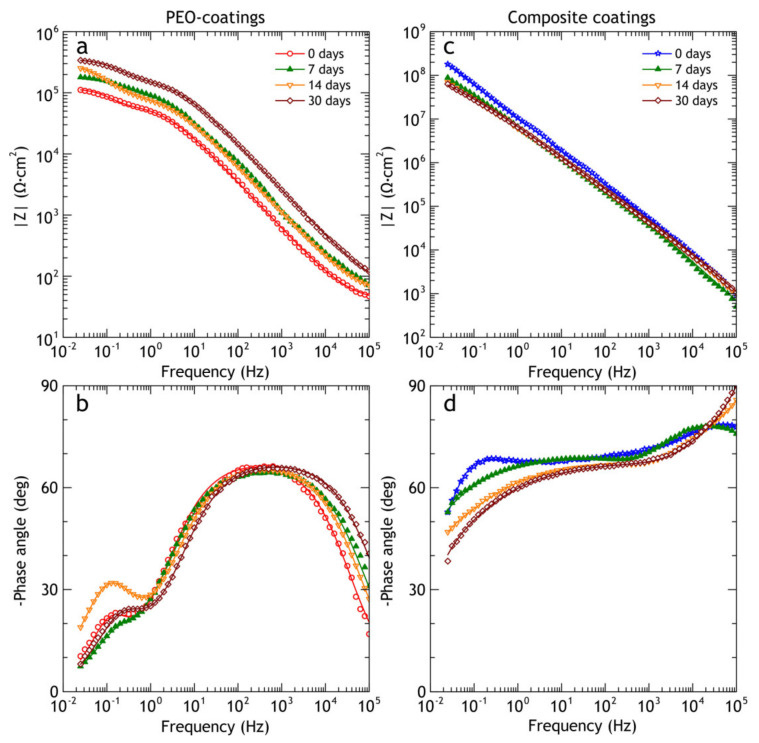
Bode plots obtained in 3% NaCl for PEO and composite coatings before and after 7, 14 and 30 days of atmospheric exposure. (**a**) PEO coatings impedance modulus (**b**) PEO coatings phase angle, (**c**) composite coatings impedance modulus, (**d**) composite coatings phase angle.

During the atmospheric corrosion the shape of impedance spectra of the PEO-coating does not change significantly (Figure 14), while the impedance modulus is continuously increased over the whole frequency range, reaching |*Z*|_f = 0.02 Hz_ = 3.4 × 10^5^ Ω·cm^2^ after 30 days of exposure, while before the exposure |*Z*|_f = 0.02 Hz_ = 1.1 × 10^5^ Ω·cm^2^. The phase angle peak of the first time constant is shifted slightly to a higher frequency region, which corresponds to partial pore sealing and, therefore, changes in the coating’s capacitance. To estimate quantitatively inner and outer layer properties the impedance spectra were fitted using EECs and EEC fit parameters are tabulated in Table 8. For PEO-coating, the *R*_1_ value that represents the resistance of the outer porous layer is increased from 6.2 × 10^4^ Ω·cm^2^ before the exposure to 1.7 × 10^5^ Ω·cm^2^ after 30 days of atmospheric exposure. The resistance of the inner barrier layer (*R*_2_) behaves similarly: it increased from 6.6 × 10^4^ Ω·cm^2^ to 1.9 × 10^5^ Ω·cm^2^. Both phenomena can be explained by the sealing of pores with corrosion products, which results in higher overall resistance. Parameters, representing capacitance of the coating (*Q*_1_ and *Q*_2_) are decreased because the sealing increases the effective thickness of the coating, which results in a shift of the phase angle peak at ≈10^3^ Hz to the higher frequency range. The *n*_1_ and *n*_2_ are almost constant, causing no significant changes in the shape of Bode plots over time. Overall, the coating barrier properties are improved after short atmospheric exposure, such behavior is often observed for conventional anodic coating, aged in a relatively low-corrosivity environment [65,66] (without sufficient ingress of chloride-saturated solution into the coating).

Before the exposure, the composite coating behaves like a capacitor with high capacitance and very small leakage current (high resistance in a parallel *R*-CPE circuit). After outdoor exposure, the CPE parameters *Q*_1_, *Q*_2_, *Q*_3_ increased from 9.4 × 10^−9^, 2.4 × 10^−8^, 1.5 × 10^−8^ Ω^−1^·cm^−2^ s^n^ to 7.82 × 10^−9^, 4.8 × 10^−8^, 7.8 × 10^−8^ Ω^−1^·cm^−2^ s^n^, respectively. This increase leads to a significant shift of phase angle peak to a lower frequency. Resistances *R*_1_, *R*_2_, *R*_3_, in contrast, decreased from 4.0 × 10^5^, 7.5 × 10^7^, 4.3 × 10^8^ Ω·cm^2^ to 3.8 × 10^4^, 3.2 × 10^7^, 1.0 × 10^8^ Ω·cm^2^, respectively. Despite the changes in impedance modulus and capacitive behavior, coating barrier properties (|Z| at low frequency) still outperform PEO-coating by two orders of magnitude and bare alloy by three orders of magnitude. One of the main driving forces for polymer degradation in a natural environment is UV radiation and it can be responsible for the appearance of micro-defects in the polymer layer because the atmospheric exposure was performed in summertime (July–August), during the maximal UV and total solar radiation throughout the season.

**Figure 15:**
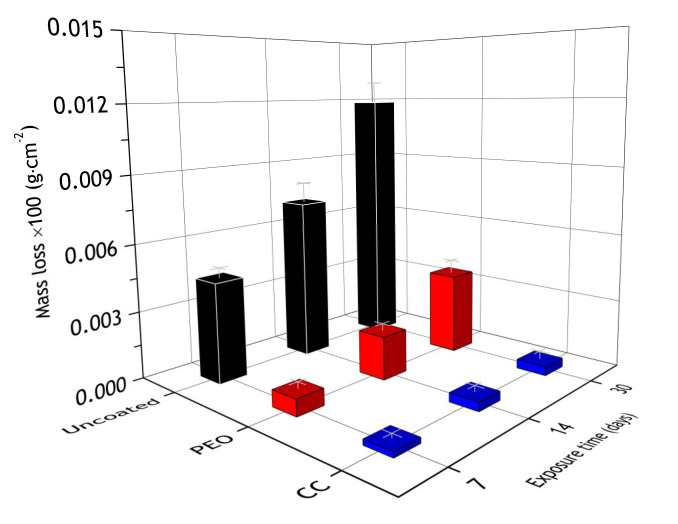
Mass loss of the specimens after 7, 14 and 30 days of atmospheric exposure.

During the atmospheric corrosion, the corrosion spots appeared at the surface of the bare aluminum alloy, and the maximum mass loss was registered for these samples, while coatings significantly reduced the mass loss (Figure 15). The observed discrepancy of results obtained for the bare alloy in comparison to the coated samples is affected by the fact that corrosion products were removed from the bare alloy by pickling. Si was detected at the PEO-coating’s surface region. Presumably, it originates from the atmospheric dust incorporated into the pores. Nevertheless, according to obtained results, its content is insufficient to lead to mass gain. In the case of the CC—its pores are almost completely sealed with the polymer and it shows no signs of silicon and a noticeable change of mass. The mass loss is associated with removing corrosion products from the surface of the coated samples being thoroughly washed before weighting.

### 3.3. Marine Corrosion

To evaluate the protective properties of a coating in extremely harsh conditions, the marine immersion in the Sea of Japan was used. Testing was performed in July–August; during these months the water temperature is relatively high (up to 24.5 °C), which leads to high activity of living organisms and high corrosion rates. Samples were withdrawn and studied in the same way as after the atmospheric corrosion test.

**Figure 16:**
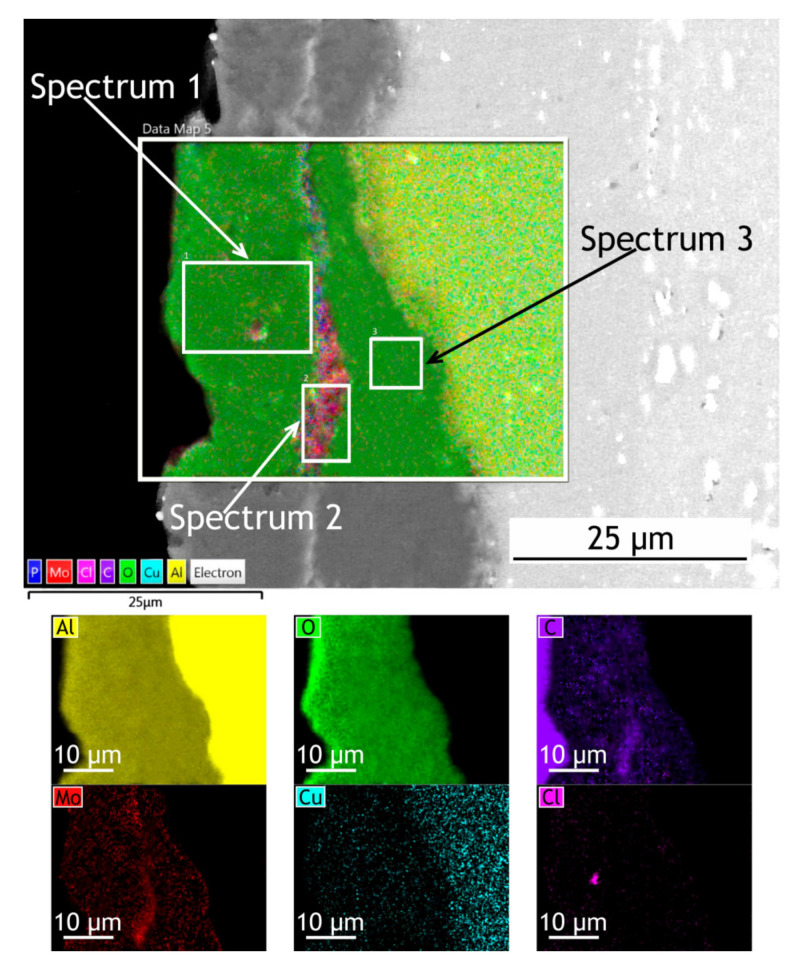
SEM image and EDX maps of the PEO-coating’s cross-section after 30 days of marine immersion.

**Figure 17:**
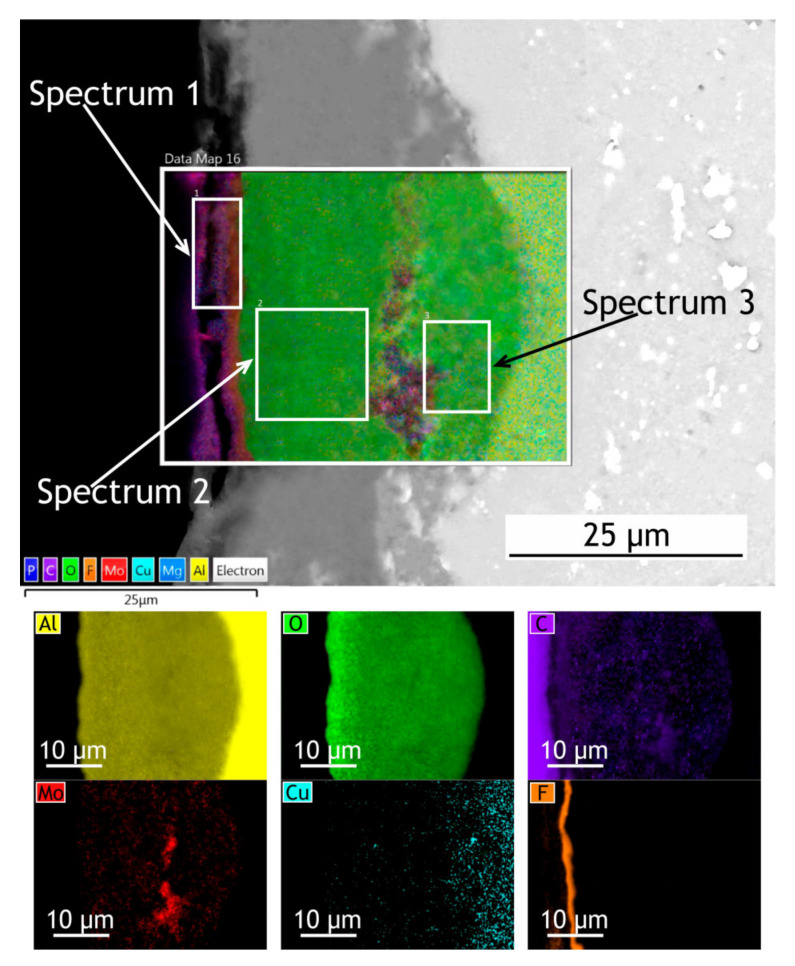
SEM image and EDX maps of the CC’s cross-section.

The exposure to the marine environment caused pitting and general corrosion at the surface of both bare alloy and PEO-coating. The CC coating remained almost intact, as it can be seen in cross-section SEM images (Figure 16 and Figure 17). Table 9 represents the elemental composition at different locations across the coatings’ thickness after 30 days of marine immersion.

After marine immersion, we also do not see any major changes in chemical composition in the coatings’ cross-section (Table 9). There is some chlorine incorporated in the outer layer of PEO-coating (Figure 16, spectrum 1 in Table 9), while there is none in the composite coating, as polymer sealing prevents ingress of corrosion species into the coating. High content of the fluorine (Figure 17), up to 30.6 wt. % in the top layer, indicates that the polytetrafluoroethylene-containing layer is not damaged and provides a high level of protective properties in a very corrosive marine environment.

**Figure 18:**
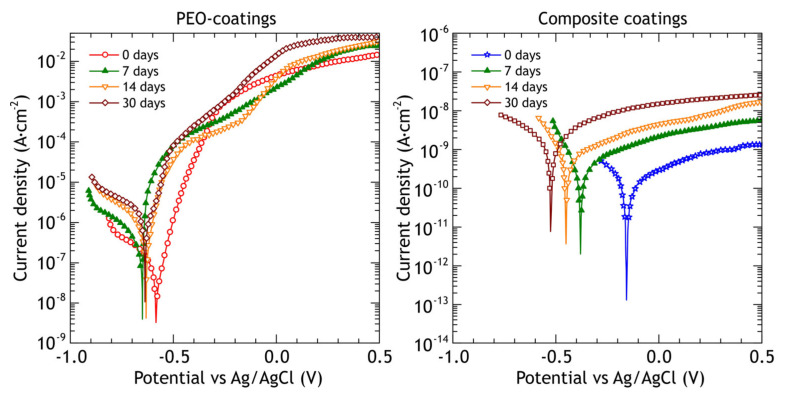
Potentiodynamic polarization curves obtained in 3% NaCl for PEO and composite coatings before and after 7, 14 and 30 days of marine immersion.

The most distinguishing feature of PEO-coatings polarization curves (Figure 18) after marine immersion is a gradual increase in cathodic branch slope *β*_c_, reaching 245 mV after 30 days, while the anodic branch slope *β*_a_ is almost constant. The corrosion potential *E*_c_ (Table 10) becomes less noble by 133 mV after the first 7 days, and then it is almost constant. Corrosion current density *j*_c_ is increased over the whole exposure period and becomes one order of magnitude higher than it was before the immersion with the corresponding decrease in impedance modulus and polarization resistance values. This indicates that PEO-coating during the marine immersion, contrary to the atmospheric exposure, does not provide significant corrosion protection. As for composite coatings, the cathodic branch slope *β*_c_ has not changed significantly during the exposure while the corrosion current density *j*_c_ increased by two orders of magnitude after 7 days of exposure and remained at the same level after 14 and 30 days. Polarization resistance *R*_p_ decreased by two orders of magnitude and remained at the same level. Even though there is a decline in polarization resistance and a rise in corrosion current density, protective properties of the composite coating after marine immersion outperform protective properties of PEO-coating before exposure by two orders of magnitude: both polarization resistance (higher) and corrosion current density (lower).

**Figure 19:**
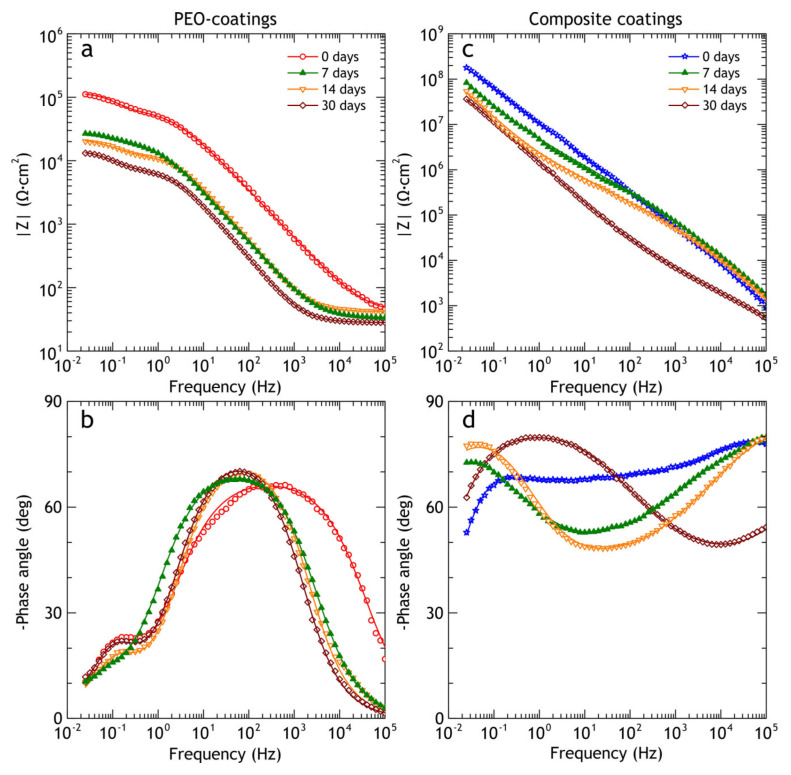
Bode plots obtained in 3% NaCl for PEO (**a**,**b**) and composite coatings (**c**,**d**) before and after 7, 14 and 30 days of marine immersion.

Impedance modulus of PEO-coating after immersion (see Figure 19) decreases in the whole frequency range. The decrease is about eightfold down to 1.4 × 10^4^ Ω cm^2^, while for the composite coating it was a twentyfive fold decrease as compared to the specimen before the test. Nevertheless, the impedance modulus of CC remained more than two orders of magnitude higher (7.2 × 10^6^ Ω cm^2^) compared to PEO-coating. EEC parameters of PEO and composite coating are tabulated in Table 11.

After the electrochemical tests, the appearance of the surface of the samples subjected to the atmospheric exposure and marine immersion has not changed except for the occurrence of single pits.

**Figure 20:**
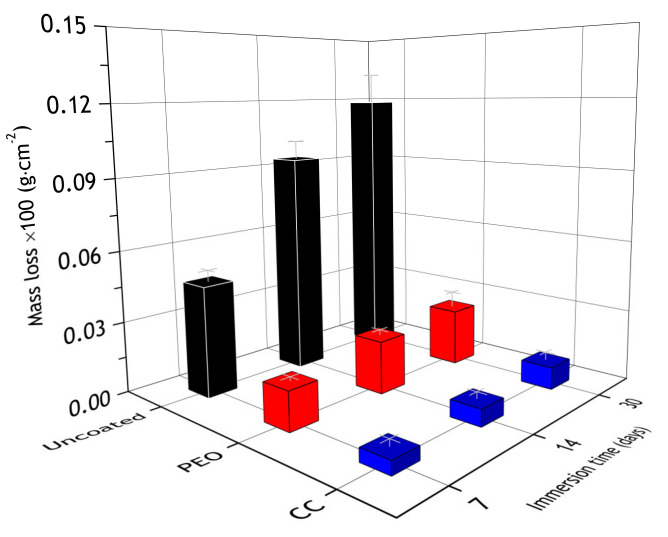
Mass loss of bare alloy, PEO and composite coated samples after 7, 14 and 30 days of marine immersion.

**Figure 21:**
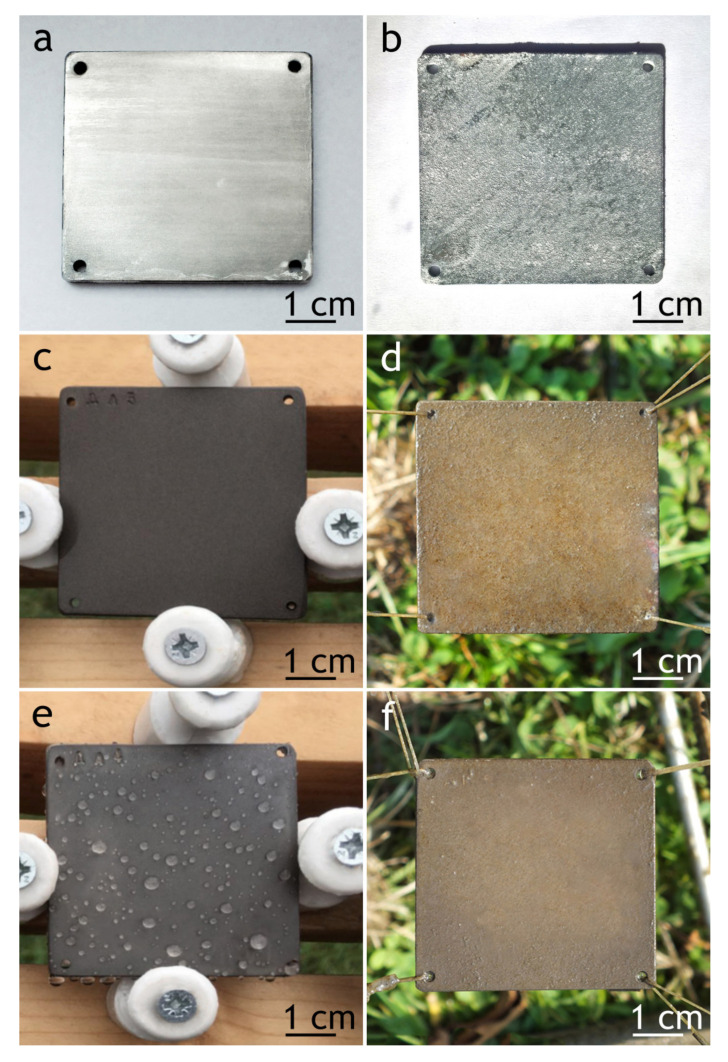
Bare alloy (**a**,**b**), PEO (**c**,**d**) and composite coatings (**e**,**f**) after 30 days of atmospheric exposure and marine immersion, correspondingly.

Although protective properties, evaluated by the electrochemical measurements, decreased, PEO and composite coatings mass loss was 12 and 220 times less compared to bare alloy (Figure 20). Additionally, there were single corrosion spots on the PEO-sample and none on the composite coating, while the surface of the bare alloy was severely corroded (Figure 21), which suggests that even though electrochemical data show some decrease in barrier properties, the surface of the of composite coating is intact. To further investigate the changes in coatings properties at the metal–oxide interface, a scratch test was performed on samples after atmospheric exposure and marine immersion for 30 days.

### 3.4. Scratch Test of Coatings after Atmospheric Exposure and Marine Immersion

**Figure 22:**
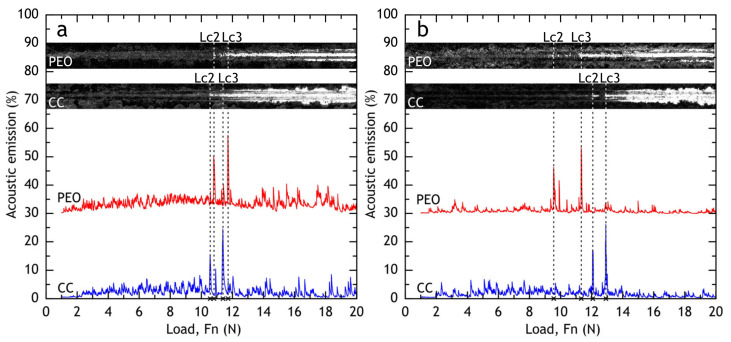
Micrograph of a full scratch, critical loads, and acoustic emission of PEO and composite coatings after 30 days of atmospheric exposure (**a**) and marine immersion (**b**).

**Figure 23:**
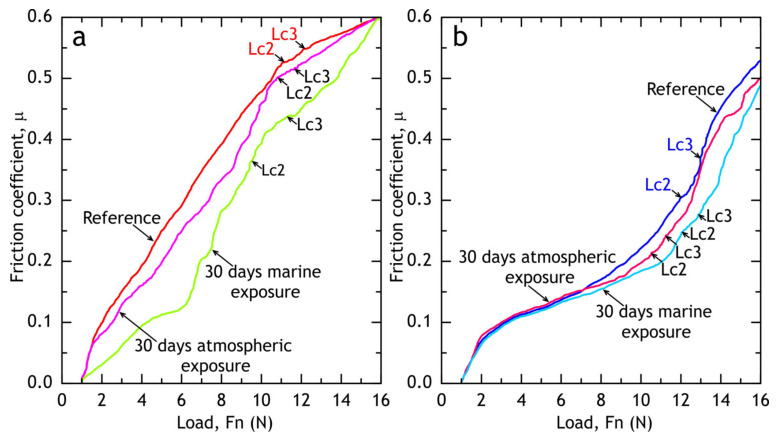
PEO (**a**) and composite (**b**) coatings’ friction coefficients after atmospheric exposure and marine immersion.

After 30 days of exposure, the samples were subjected to scratch tests (see Figure 22 and Figure 23) and critical loads (*L*_c_) and the friction coefficient was measured. Although barrier properties of PEO-coatings measured by the electrochemical methods after atmospheric corrosion were high, a degradation of the coating’s mechanical properties is evident, which is caused by corrosion initiation sites at the oxide–metal interface. Composite coatings showed no sign of mechanical properties degradation after atmospheric exposure as critical loads did not reduce compared to the reference samples (Figure 9). We suggest that the observed decrease in friction coefficient can be attributed to loose corrosion products and additionally to the biofilm, formed on the samples after the marine immersion.

## 4. Conclusions

Polymer-containing composite coating fabrication on the PEO layer base is a promising technology for corrosion protection and providing functional properties, such as a low friction coefficient.

Atmospheric exposure of the PEO-coating leads to partial sealing of pores, increasing its barrier properties, ascertained by EIS and potentiodynamic polarization, although the corrosion initiation sites at the metal-oxide interface lowered adhesion of PEO-coating to the substrate. As for composite coating, despite some degradation of barrier properties due to UV radiation, adhesion remained constant and coating barrier properties (impedance modulus at low frequency) still outperformed PEO-coating by two orders of magnitude and bare D16 alloy by three orders of magnitude.

Marine corrosion caused some degradation of electrochemical properties of PEO and composite coatings, but composite coatings had better adhesion and barrier properties, compared to PEO-coatings. Although the decrease in CC barrier properties was observed, the impedance modulus at low frequency remained more than two orders of magnitude higher compared to reference PEO samples, and although the corrosion initiation sites at the metal-oxide interface lowered adhesion of PEO-coating to the substrate, adhesion remained constant.

Electrochemical impedance spectroscopy and potentiodynamic polarization measurements revealed the differences in coating behaviors during the atmospheric exposure: PEO-coating increased its barrier properties due to partial sealing of pores; the composite coating exhibited some degradation of protective properties due to UV radiation. Nevertheless, barrier properties (impedance modulus at low frequency) of the composite coating still outperformed PEO-coating by two orders of magnitude and bare D16 alloy by three orders of magnitude.

Scratch test of PEO coatings showed that there is some degradation of the coating’s mechanical properties, which is caused by corrosion initiation sites at the oxide-metal interface and could not be seen visually or measured by electrochemical tests, which only show an increase in barrier properties due to partial pore sealing. Composite coatings showed no sign of mechanical properties degradation after atmospheric exposure as critical loads did not reduce compared to the reference samples.

Marine corrosion caused a decrease in electrochemical properties of PEO and composite coatings, but composite coating had better adhesion and barrier properties, compared to PEO-coating. Although the decrease in CC barrier properties was observed, the impedance modulus at low frequency remained more than two orders of magnitude higher compared to reference PEO samples.

## Figures and Tables

**Table 1:** Elemental composition of the coatings (Figure 3).

PEO				CC			
Spectrum 1		Spectrum 2		Spectrum 1		Spectrum 2	
Element	wt. (%)	Element	wt. (%)	Element	wt. (%)	Element	wt. (%)
O	45.0	Mo	70.8	O	45.2	O	31.3
Al	33.0	O	21.5	Al	31.2	Al	29.0
C	11.7	Al	5.0	C	19.1	C	25.3
Mo	4.8	P	2.7	F	2.6	F	10.5
P	3.3	-	-	Mo	1.9	Mo	3.3
Na	1.7	-	-	-	-	P	0.6
K	0.5	-	-	-	-	-	-

**Table 2:** Elemental composition at different locations across coatings’ thickness (Figure 4 and Figure 5).

PEO						CC					
Top (Spectrum 1)		Middle (Spectrum 2)		Bottom (Spectrum 3)		Top (Spectrum 1)		Middle (Spectrum 2)		Bottom (Spectrum 3)	
Element	wt. (%)	Element	wt. (%)	Element	wt. (%)	Element	wt. (%)	Element	wt. (%)	Element	wt. (%)
O	54.3	O	52.6	O	51.4	O	33.4	O	57.7	O	51.2
Al	38.3	Al	32.2	Al	35.3	C	29.5	Al	33.4	Al	32.6
C	5.2	C	12.2	C	5.6	F	20.2	C	6.3	C	8.1
Mo	1.3	Mo	1.9	Mo	4.3	Al	14.8	Mo	2.6	Mo	4.2
P	0.9	P	0.6	P	1.8	Mo	2.1	Cu	0.6	P	2.8
-	-	Cu	0.5	Cu	1.2	-	-	-	-	Cu	1.1
-	-	-	-	Mg	0.4	-	-	-	-	-	-

**Table 3:** Electrochemical parameters of the bare alloy, PEO and composite coatings (Figure 6).

Sample	*β*_a_ (mV)	−*β*_c_ (mV)	*j*_c_ (A·cm^−2^)	*E*_c_ (mV)	*R*_p_ (Ω cm^2^)	|*Z*|_f = 0.02 Hz_ (Ω cm^2^)
Bare alloy	307	211	4.5 × 10^−6^	−757	1.2 × 10^4^	1.1 × 10^4^
PEO-coating	65	208	1.2 × 10^−7^	−583	1.8 × 10^5^	1.1 × 10^5^
Composite coating	174	80	1.1 × 10^−10^	−160	2.2 × 10^8^	1.8 × 10^8^

**Table 4:** Surface elemental composition near the pits formed after the potentiodynamic polarization (Figure 7).

PEO				CC			
Spectrum 1		Spectrum 2		Spectrum 1		Spectrum 2	
Element	wt. (%)	Element	wt. (%)	Element	wt. (%)	Element	wt. (%)
Al	38.8	O	49.7	C	42.6	F	31.2
O	38.1	Al	35.1	F	27.2	C	26.5
Cu	13.2	C	11.2	O	20.6	Al	20.8
C	8.5	Cu	1.4	Al	7.3	O	20.3
Cl	1.3	Mo	1.3	Mo	1.8	Mo	1.2
-	-	Cl	1.0	Cl	0.6	-	-

**Table 5:** Bare alloy, PEO and composite coatings equivalent electrical circuit (EEC) parameters (Figure 8).

Sample	CPE_1_		*R*_1_ (Ω·cm^2^)	CPE_2_		*R*_2_ (Ω·cm^2^)	W			CPE_3_		*R*_3_ (Ω·cm^2^)
	*Q*_1_ (Ω^−1^·cm^−2^ s^n^)	*n*_1_		*Q*_2_ (Ω^−1^·cm^−2^ s^n^)	*n*_2_		*R*_W_ (Ω·cm^2^)	T (s)	P	*Q*_3_ (Ω^−1^·cm^−2^ s^n^)	*n*_3_	
Bare alloy	–	–	–	6.4 × 10^−5^	0.90	4.2 × 10^3^	2.8 × 10^3^	1.15	0.32	–	–	–
PEO-coating	2.0 × 10^−6^	0.77	6.2 × 10^4^	2.5 × 10^−5^	0.79	6.6 × 10^4^	–	–	–	–	–	–
Composite coating	9.4 × 10^−9^	0.86	4.0 × 10^5^	2.4 × 10^−8^	0.60	7.5 × 10^7^	–	–	–	1.5 × 10^−8^	0.86	4.3 × 10^8^

**Table 6:** Elemental composition at different locations across coatings’ thickness after 30 days of atmospheric exposure (Figure 11 and Figure 12).

PEO						CC					
Top (Spectrum 1)		Middle (Spectrum 2)		Bottom (Spectrum 3)		Top (Spectrum 1)		Middle (Spectrum 2)		Bottom (Spectrum 3)	
Element	wt. (%)	Element	wt. (%)	Element	wt. (%)	Element	wt. (%)	Element	wt. (%)	Element	wt. (%)
O	48.7	O	55.8	O	48.8	O	38.8	O	55.0	O	42.5
Al	33.9	Al	43.1	Al	38.0	Al	18.2	Al	37.4	Al	35.8
C	8.4	Mo	0.6	C	6.7	C	21.4	C	5.6	C	9.7
Si	7.1	P	0.5	Mo	4.9	F	20.9	Mo	1.6	F	8.1
Mo	1.2	-	-	P	1.6	Mo	0.7	P	0.4	Mo	3.9
P	0.7	-	-	-	-	-	-	-	-	-	-

**Table 7:** Electrochemical parameters of PEO and composite coatings before and after 7, 14 and 30 days of atmospheric exposure (Figure 13).

Coating	Exposure, Days	*β*_a_ (mV)	−*β*_c_ (mV)	*j*_c_ (A·cm^−2^)	*E*_c_ (mV)	*R*_p_ (Ω·cm^2^)	|*Z*|_f = 0.02 Hz_, (Ω cm^2^)
PEO	0	65	208	1.2 × 10^−7^	−583	1.8 × 10^5^	1.1 × 10^5^
	7	72	143	5.7 × 10^−8^	−572	3.6 × 10^5^	1.8 × 10^5^
	14	62	145	4.2 × 10^−8^	−569	4.5 × 10^5^	2.5 × 10^5^
	30	56	190	3.9 × 10^−8^	−576	4.8 × 10^5^	3.4 × 10^5^
CC	0	174	80	1.1 × 10^−10^	−160	2.2 × 10^8^	1.8 × 10^8^
	7	234	89	3.2 × 10^−10^	−225	8.8 × 10^7^	8.3 × 10^7^
	14	243	95	5.3 × 10^−10^	−327	5.7 × 10^7^	5.4 × 10^7^
	30	255	102	7.6 × 10^−10^	−441	4.2 × 10^7^	3.6 × 10^7^

**Table 8:** PEO and composite coatings EEC parameters before and after 7, 14 and 30 days of atmospheric exposure (Figure 14).

Sample	Exposure Time, Days	CPE_1_		*R*_1_ (Ω·cm^2^)	CPE_2_		*R*_2_ (Ω·cm^2^)	CPE_3_		*R*_3_ (Ω·cm^2^)
		*Q*_1_ (Ω^−1^·cm^−2^ s*^n^*)	*n*_1_		*Q*_2_ (Ω^−1^·cm^−2^ s*^n^*)	*n*_2_		*Q*_3_ (Ω^−1^·cm^−2^ s*^n^*)	*n*_3_	
PEO	0	2.0 × 10^−6^	0.77	6.2 × 10^4^	2.5 × 10^−5^	0.79	6.6 × 10^4^	-	-	
	7	1.2 × 10^−6^	0.74	1.2 × 10^5^	1.6 × 10^−5^	0.85	7.2 × 10^4^	-	-	
	14	1.3 × 10^−6^	0.75	9.3 × 10^4^	1.1 × 10^−5^	0.86	2.1 × 10^5^	-	-	
	30	5.3 × 10^−7^	0.76	1.7 × 10^5^	6.0 × 10^−6^	0.83	1.9 × 10^5^	-	-	
Composite coating	0	9.4 × 10^−9^	0.86	4.0 × 10^5^	2.4 × 10^−8^	0.60	7.5 × 10^7^	1.5 × 10^−8^	0.86	4.3 × 10^8^
	7	2.8 × 10^−9^	0.92	7.7 × 10^3^	1.6 × 10^−7^	0.44	5.1 × 10^7^	7.3 × 10^−8^	0.92	2.8 × 10^8^
	14	5.2 × 10^−9^	0.88	3.8 × 10^4^	1.9 × 10^−7^	0.51	2.1 × 10^6^	1.1 × 10^−7^	0.98	1.7 × 10^8^
	30	4.2 × 10^−9^	0.84	3.1 × 10^3^	0.7 × 10^−7^	0.52	2.0 × 10^6^	1.2 × 10^−7^	0.79	1.0 × 10^8^

**Table 9:** Elemental composition at different locations across coatings’ thickness after 30 days of marine immersion (Figure 16 and Figure 17).

PEO						CC					
Top (Spectrum 1)		Middle (Spectrum 2)		Bottom (Spectrum 3)		Top (Spectrum 1)		Middle (Spectrum 2)		Bottom (Spectrum 3)	
Element	wt. (%)	Element	wt. (%)	Element	wt. (%)	Element	wt. (%)	Element	wt. (%)	Element	wt. (%)
O	53.5	O	48.8	O	54.2	O	25.4	O	54.1	O	46.4
Al	39.2	Al	35.6	Al	38.6	Al	8.7	Al	36.7	Al	39.9
C	5.4	C	11.4	C	3.9	C	34.4	C	4.7	C	8.8
Mo	1.3	Mo	3.9	Mo	1.7	Mo	0.9	Mo	3.4	Mo	3.5
P	0.4	P	0.3	P	1.3	F	30.6	P	0.7	Cu	0.6
Cl	0.2			Cu	0.3			Mg	0.4	Mg	0.5

**Table 10:** Electrochemical parameters of PEO and composite coatings before and after 7, 14 and 30 days of marine immersion (Figure 18).

Sample	Exposure Time, Days	*β*_a_ (mV)	−*β*_c_ (mV)	*j*_c_ (A·cm^−2^)	*E*_c_ (mV)	*R*_p_ (Ω·cm^2^)	|*Z*|_f = 0.02 Hz_, (Ω cm^2^)
PEO	0	65	208	1.2 × 10^−7^	−583	1.8 × 10^5^	1.1 × 10^5^
	7	60	215	3.8 × 10^−7^	−650	3.8 × 10^4^	5.6 × 10^4^
	14	58	220	6.5 × 10^−7^	−644	3.1 × 10^4^	3.0 × 10^4^
	30	60	245	1.2 × 10^−6^	−646	1.8 × 10^4^	1.4 × 10^4^
Composite coating	0	174	80	1.1 × 10^−10^	−160	2.2 × 10^8^	1.8 × 10^8^
	7	59	406	2.5 × 10^−10^	−384	9.0 × 10^7^	8.7 × 10^7^
	14	63	440	5.8 × 10^−10^	−451	4.1 × 10^7^	2.7 × 10^7^
	30	72	314	1.3 × 10^−9^	−583	2.0 × 10^7^	7.2 × 10^6^

**Table 11:** PEO and composite coatings EEC parameters before and after 7, 14 and 30 days of marine immersion (Figure 19).

Sample	Exposure Time, Days	CPE_1_		*R*_1_ (Ω·cm^2^)	CPE_2_		*R*_2_ (Ω·cm^2^)	CPE_3_		*R*_3_ (Ω·cm^2^)
		*Q*_1_ (Ω^−1^·cm^−2^ s^n^)	*n*_1_		*Q*_2_ (Ω^−1^·cm^−2^ s^n^)	*n*_2_		*Q*_3_ (Ω^−1^·cm^−2^ s^n^)	*n*_3_	
PEO	0	2.0 × 10^−6^	0.77	6.2 × 10^4^	2.5 × 10^−5^	0.79	6.6 × 10^4^	-	-	-
	7	1.5 × 10^−5^	0.92	1.8 × 10^4^	3.7 × 10^−4^	0.71	1.5 × 10^4^	-	-	-
	14	1.3 × 10^−5^	0.88	1.5 × 10^4^	1.2 × 10^−4^	0.67	5.5 × 10^4^	-	-	-
	30	1.3 × 10^−5^	0.89	2.5 × 10^4^	7.2 × 10^−5^	0.74	1.1 × 10^5^	-	-	-
CC	0	9.4 × 10^−9^	0.86	4.0 × 10^5^	2.4 × 10^−8^	0.60	7.5 × 10^7^	1.5 × 10^−8^	0.86	4.3 × 10^8^
	7	2.8 × 10^−9^	0.92	7.7 × 10^3^	1.6 × 10^−7^	0.44	5.1 × 10^7^	7.3 × 10^−8^	0.92	2.7 × 10^8^
	14	5.2 × 10^−9^	0.88	3.8 × 10^4^	1.9 × 10^−7^	0.51	2.1 × 10^6^	1.1 × 10^−7^	0.98	5.7 × 10^7^
	30	4.2 × 10^−9^	0.84	3.1 × 10^3^	0.7 × 10^−7^	0.52	2.0 × 10^6^	1.2 × 10^−7^	0.79	2.3 × 10^7^
